# Sex-biased gene expression and recent sex chromosome turnover

**DOI:** 10.1098/rstb.2020.0107

**Published:** 2021-09-13

**Authors:** Nicolás Lichilín, Athimed El Taher, Astrid Böhne

**Affiliations:** ^1^ Zoological Institute, Department of Environmental Sciences, University of Basel, Vesalgasse 1, 4051 Basel, Switzerland; ^2^ Center for Molecular Biodiversity Research, Zoological Research Museum Alexander Koenig, Adenauerallee 160, 53113 Bonn, Germany

**Keywords:** sex chromosome turnover, sex-biased gene expression, sexual conflict, cichlid fishes

## Abstract

Cichlids are well known for their propensity to radiate generating arrays of morphologically and ecologically diverse species in short evolutionary time. Following this rapid evolutionary pace, cichlids show high rates of sex chromosome turnover. We here studied the evolution of sex-biased gene (SBG) expression in 14 recently diverged taxa of the Lake Tanganyika Tropheini cichlids, which show different XY sex chromosomes. Across species, sex chromosome sequence divergence predates divergence in expression between the sexes. Only one sex chromosome, the oldest, showed signs of demasculinization in gene expression and potentially contribution to the resolution of sexual conflict. SBGs in general showed high rates of turnovers and evolved mostly under drift. Sexual selection did not shape the rapid evolutionary changes of SBGs. Male-biased genes evolved faster than female-biased genes, which seem to be under more phylogenetic constraint. We found a relationship between the degree of sex bias and sequence evolution driven by sequence differences among the sexes. Consistent with other species, strong sex bias towards sex-limited expression contributes to resolving sexual conflict in cichlids.

This article is part of the theme issue ‘Challenging the paradigm in sex chromosome evolution: empirical and theoretical insights with a focus on vertebrates (Part II)’.

## 1. Introduction

Males and females of the same species share most of their genome but differ in their fitness optima for morphological, physiological as well as behavioural traits [1,2]. This lays ground for (widespread) sexual conflict across the genome [3]. Fixed differences between the sexes in species with genetic sex determination can be as little as a single base pair [4] but can also encompass entire chromosomes (i.e. [5]), the so-called sex chromosomes. While sex chromosomes initially define the sex of an individual (usually upon fertilization), the establishment and maintenance of sex differences throughout an individual's lifespan are mostly attributed to differential, i.e. sex-biased gene (SBG) expression of loci spread across the genome.

Owing to their sex-specific inheritance and selection, sex chromosomes in particular can be subject to feminization or masculinization of gene expression. They are thus major contributors to reaching sexual optima and hence the resolution of sexual conflict [6]. This is evidenced by an over-representation of SBGs on sex chromosomes (e.g. gene expression is feminized in male heterogametic systems on the X chromosome [7] and masculinized in female heterogametic systems on Z chromosomes [8]). Still, a large fraction of the rest of the genome also shows sex bias in gene expression in many organisms, which is commonly taken as a signature of evolution under the influence of sexual antagonism and ongoing sexual conflict (reviewed in [9]).

While it seems rather evident that SBGs often show both high rates of expression and sequence divergence, likely driven through sexual selection, it is less clear how sex chromosome turnovers impact SBG expression and *vice versa*.

We here investigated the evolution of SBG expression in 14 closely related fish taxa that experienced recent sex chromosome turnovers or losses. In addition, these fishes show variation in sexual dimorphism and hence probably in their degree of sexual selection. More precisely, we analysed 14 cichlid taxa of eight genera that all belong to the tribe Tropheini of the Lake Tanganyika (LT) cichlid adaptive radiation. The so-called Haplochromini, which seeded the radiations of Lakes Victoria and Malawi comprising more than 1500 species [10], are phylogenetically nested within the LT cichlid radiation [11] and Tropheini represent the LT endemic Haplochromini. The here investigated Tropheini cichlid species have diverged less than 5 million years ago (Ma) [11]. As part of the adaptive radiation of LT cichlids, they show impressive phenotypic and ecologic diversity despite short divergence times [11].

**Figure 1.**
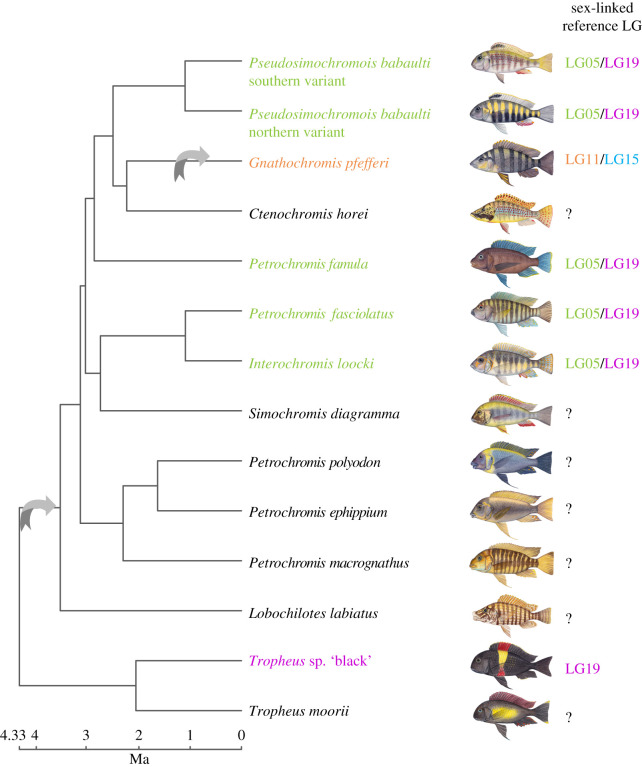
The previously described sex chromosomes of the here investigated taxa [13] are indicated next to the species name and refer to LGs of the reference genome of the Nile tilapia (*Oreochromis niloticus*). All identified sex chromosome systems were male heterogametic XY. Arrows indicate previously identified sex chromosome turnover events, i.e. changes in sex-linked LGs; species with a ‘?’ lacked signs of sex differences in the available data [13]. A time-calibrated species tree [11] was pruned to the here studied taxa. Fish drawings were created by Julie Johnson and used with permission.

Through an analysis of 38 Tropheini taxa (near complete taxon sampling, Tropheini contains 24 valid described and 16 undescribed species [12]), we previously identified three different XY sex chromosomal systems that we placed on linkage groups (LGs) of a reference genome ([13], illustrated in figure 1). There is one system with signs of sex chromosome differentiation comprising almost the full length of LG19. This system is likely the ancestral state within Tropheini with an estimated origin at approximately 4.95 Ma. From the here studied taxa, only *Tropheus* sp. ‘black’ has this XY system. In genera other than *Tropheus*, several species have an XY system located at the beginning of LG5 and the end of LG19, suggesting a fusion of these chromosomes or a larger translocation. This system is present in five of the here studied taxa (*Interochromis loocki*, *Petrochromis famula*, *Petrochromis fasciolatus*, *Pseudosimochromis babaulti* northern variant and *P. babaulti* southern variant) and evolved within Tropheini in the branch leading to all genera but *Tropheus* (estimated age approx. 3.79 Ma). In a single species, *Gnathochromis pfefferi*, the third XY system evolved, which is located on LG11 and LG15, supportive of another chromosomal fusion. For the remaining here studied species, *Ctenochromis horei*, *Simochromis diagramma*, *Petrochromis polyodon*, *Petrochromis ephippium*, *Petrochromis macrognathus*, *Lobochilotes labiatus* and *Tropheus moorii*, no sex chromosome has been identified so far. These species seemingly lost the LG5/LG19 and LG19 systems, respectively. It remains to be resolved to which—if any—sex chromosome system they transitioned to.

In general, cichlid sex chromosomes are young [13,14] and although differences are detectable on the sequence level, the gametologues are not morphologically differentiated such as the heteromorphic sex chromosomes of mammals and most birds, in which female-biased genes (FBGs) are over-represented on the X chromosome [15] and male-biased genes (MBGs) on the Z chromosome [16], respectively.

Within the here studied 14 taxa, we first characterized the amount of sex bias in gene expression in gonads and four different somatic organs (gill, liver, brain, jaw). We then investigated how sex bias in gene expression evolved with respect to changes in sex chromosomes and how this is accompanied by patterns of sequence evolution.

This dataset allowed us to investigate: (i) how fast sex chromosomes can accumulate SBG expression, (ii) how turnover of sex chromosomes impacts sex bias, and (iii) which selection regimes act on sex chromosomes and SBGs of recently diverged taxa.

## 2. Methods

### (a) Sequencing and gene expression data

We analysed Illumina TruSeq RiboZero 125 bp single-end transcriptome data from 14 LT cichlid taxa included in El Taher *et al*. [17] accessible under the BioProject accession number PRJNA552202. These data comprise typically three biological replicates per sex and organ for five different organs of adult, mature individuals captured in the wild: gonads, brain, liver, gills and lower pharyngeal jaw (here for simplicity referred to as jaw). These organ types were included in the original study owing to their potential function in ecological, physiological, and behavioural adaptations during cichlid adaptive radiations [12,18–20] and have also previously been studied in multi-organ analysis of gene expression in other species enabling comparisons [21,22]. For details on RNA-sequencing data available to us, see the electronic supplementary material, table S1.

We used count data on the gene level as described in [17]. In brief, these data were generated by mapping RNA-sequencing reads to the Nile tilapia (*Oreochromis niloticus*) reference genome assembly (ASM185804v2) and summarizing counts per annotated gene feature with HTSeq (v. 0.6.1) [23].

### (b) Differential gene expression

Gene expression was analysed with the Bioconductor R package DESeq2 (v. 1.22.2) [24] in R (v. 3.5.2 and v. 3.6). Count data were transformed with the mean of variance stabilizing transformation (VST) and subsequently one *T.* sp. ‘black’ liver sample excluded because it had a median count value close to zero indicative of sequencing failure. We then ran principal component analyses (PCAs) and sample clustering of Euclidean distances on VST values for all samples combined, for all samples of a species and per species for each organ to identify potential outlier samples. Two individuals (a ‘male’ *T.* sp. ‘black’ and a ‘female’ *I. loocki*) were excluded from all subsequent analyses since the expression of their organs showed clear clustering with individuals of the opposite sex upon an inspection of per species PCAs and Euclidean distance analyses. We further could not analyse gene expression of liver samples in *G. pfefferi* and *C. horei* owing to a lack of sufficient replicates of each sex in the original study [17] nor in *I. loocki* owing to a lack of female replicates after removing the individual mentioned above. We further excluded a single liver sample of a male *P. babaulti* northern variant since upon an inspection of the PCA of all samples together, this sample did not group with other liver samples. Details on initially available and subsequently included samples are listed in the electronic supplementary material, table S1.

The final dataset with typically three (requiring a minimum of two) replicates per organ and sex and species was composed of 384 samples. Genes with count data of less than 1 across all samples were removed resulting in 34 925 genes out of 38 425. Comparison of Euclidian distances of VST-transformed expression values within and between species showed consistently lower values for biological replicates within a species than between species (electronic supplementary material, figure S1*a*). A comparison of the variance of expression calculated on trimmed median of means (TMM) expression values (implemented in edgeR (v. 3.24.3 [25,26]) per gene and sex and organ showed the same pattern (electronic supplementary material, figure S1*a*).

Differential expression between sexes was computed with DESeq2 per species and organ using sex as model contrast with the *independent hypothesis weighting* method of the R package IHW (v. 1.10.1) [27] to correct for multiple testing with a weighted Benjamini and Hochberg method which shows increased detection power [27]. SBGs were defined as genes with an adjusted *p*-value < 0.05 and an absolute log2-fold change of expression (LFC) value greater than 2. Categories of sex-biased expression were determined based on the LFC distribution across samples and organs with low = LFC > 2 and LFC < 2.6; mid = LFC ≥ 2.6 and LFC < 4.1; high = LFC ≥ 4.1 and LFC < 6.5; extreme = LFC ≥ 6.5.

To investigate differences in variance across taxa, we tested if the number of SBGs identified per species and organ was dependent on the gene expression variance within replicates (calculated as the median of all variances of the TMM normalized counts per gene and sex within each organ). We did not find any correlation across all samples together (*ρ* = 0.24, *p* = 0.4; electronic supplementary material, figure S1*b*) neither within organs (electronic supplementary material, figure S1*b*) confirming the validity of the used approach to detect between species differences in SBG expression.

To determine fractions of shared SBGs, we compared the sets of MBGs and FBGs across taxa in R with the package UpsetR (v. 1.4.0) [28].

Functional gene annotation was done within Blast2Go (v. 5.2.5) [29] based on an annotation file generated for the reference genome. The most significant gene ontology terms were obtained by setting a false discovery rate of less than 0.05. These terms were subsequently visualized with REVIGO [30].

### (c) Statistical analyses for genes in expression categories and on linkage groups

Gene locations on LGs are based on the annotation file of the reference genome of the Nile tilapia. For statistical analysis across groups, we first tested for fits to normal distribution with the Shapiro–Wilk test and for the homogeneity of variances with the Leven's test in the car package [31] in R. Comparisons were done with non-parametrical statistical tests, for comparisons across gene categories with a Kruskal−Wallis test and a two-sample Wilcoxon test (also known as the Mann–Whitney test) as *post hoc* test. For comparisons of autosomes to sex chromosomes for the amount of SBGs and comparisons of population statistics, we applied a Wilcoxon test. Visualization of the statistical tests was done in R with the package ggpubr (v. 0.4.0) (https://rpkgs.datanovia.com/ggpubr/). To test for a chromosomal feminization in gonads within a species across LGs, the difference between the number of MBGs and FBGs was computed and a one-sided Fisher's exact test was calculated for each LG within a species.

### (d) Sequence and expression evolution

Calculations were done based on mapped RNA-data derived from [17] followed by marking duplicate reads with Picard (v. 2.9.2) (https://broadinstitute.github.io/picard/), splitting reads with Ns in the cigar string into multiple alignments, hard clipping of mismatching overhangs, followed by base quality score recalibration and indel realignment with GATK (v. 3.7) based on the GATK Best Practices recommendations [32,33]. Individual files were merged and sorted with SAMtools (v. 1.7) [34].

Intersex Fst, Tajima's *D* and nucleotide diversity per species were computed with ANGSD [35] as previously described [36], accounting for sequencing uncertainty and the unevenness in sequencing depth from transcriptomic data.

In brief, we removed reads that did not map uniquely and/or had a mapping quality below 20. Sites were kept if they were present in at least three individuals, had a minimum base quality score of 13 and a minimum total depth of 3. Sample allele frequencies were calculated from likelihoods at each site from genotype likelihoods with the SAMtools model. We estimated the overall folded site frequency spectrum (SFS) in the absence of the ancestral state information. Next, we proceeded to compute genetic diversity indices using the SFS as prior information for each nucleotide position. Estimations per gene were done based on the position in the genome annotation file in R. To obtain accurate estimates of nucleotide diversity (*π*), we corrected *π* by the number of variants and invariant sites covered within a gene as suggested in [37].

For intersex Fst, we additionally removed bases with coverage in less than two individuals per sex. The overall unfolded SFS for the population was computed and Hudson's Fst was calculated per site, as described in [36] and summarized per gene in R. For each gene, we computed the ratio between the sum of the *α* and the sum of *α* + *β* from [38] as in [39], where per gene Fst is the ratio between the sum of the between-populations (in our case between-sexes) variance across loci and the sum of the total variance across loci. This results in per gene estimates of Fst shrunken towards the genome-wide Fst reducing noise and delivering more accurate estimates [39]. The Fst estimations applied use a method of moment estimators, which can result in negative values as described in [40].

### (e) Analysis of selection

Rates of non-synonymous to synonymous substitutions per gene and species were assessed as described in [11] for the subset of taxa investigated here by estimating for each gene the synonymous and non-synonymous substitutions in each species by pairwise comparisons to the orthologous Nile tilapia sequence with codeml in runmode −2 within PAML (v. 4.9e) [41]. Selection on gene expression levels of transcript per million (TPM) normalized values was assessed following the calculation of Δ*x* as described in [42] and applied to similar datasets previously (e.g. [43,44]). Δ*x* was calculated for each focal species with *T. moorii* as the reference species and proxy for ancestral expression levels as the most basal branching species in our species tree. For *T. moorii* as the focal species, we used *T.* sp. ‘black’ as reference species.

### (f) Gene expression phylogenies

Gene expression trees were calculated on pairwise distance matrices obtained from normalized gene counts after TMM normalization as previously described for mammals [22]. We calculated the mean expression values for each species and organ. Distances between species were computed as 1 − *ρ*, where *ρ* is Spearman's correlation coefficient. Expression phylogenies were reconstructed with the R package ape (v. 5.3) [45] with bootstrap resampling reliability with 1000 iterations. Differences in the total branch length from root to tips were assessed with a Kruskal–Wallis test and Wilcoxon *post hoc* tests across each category of sex-biased and unbiased genes. We calculated Robinson–Foulds (RF) topology distance in R with phangorn (v. 2.5.5) [46,47] in comparison to a species tree [11] pruned to the here studied taxa.

### (g) Ancestral reconstruction of sex-biased gene expression

To investigate changes in SBGs across the species tree, we reconstructed ancestral sex bias with the *ace* function in the ape package in R based on categorical information for each gene (i.e. FBG, MBG or unbiased derived from differential expression analysis described above), under maximum-likelihood (ML) estimation with an equal-rates model using joint estimation procedures that take into account all the information for each node.

We assessed the status of sex bias for every gene at every internal node and calculated the changes in bias in R along the branches. Visualization was done with the R package ggtree (v. 1.16.6) [48,49].

### (h) Correlations of sex bias to traits

Association of traits with the total number of FBG and MBG turnovers on terminal branches was calculated with the R package nlme (v. 3.1-148) (http://CRAN.R-project.org/package=nlme). We fitted a linear model using generalized least squares under ML phylogenetically controlled using the species tree topology (see above) for sperm competition rank, degree of sexual dimorphism (kindly provided by Adrian Indermaur, an outstanding expert in cichlid biology) and the degree of sex chromosome differentiation derived from [13]. Sperm competition rank uses a composite score that combines behavioural, ecological and within-brood paternity information and was derived from [50,51]. All trait values are listed in the electronic supplementary material, table S2.

### (i) Gene expression evolution

Pairwise Spearman's *ρ* correlation coefficients of gene expression for each species, sex and organ were correlated with pairwise divergence times obtained from the time-calibrated species tree of [11] with the R package ape as described in [17].

To test if genes on sex chromosomes evolved faster, we calculated Spearman's *ρ* correlation coefficient of TMM expression values of count data per LGs as previously suggested [52]. In brief, we calculated Spearman's *ρ* correlation coefficient between taxa based on the mean male and female TMM normalized count values per gene, separately for each LG. Afterwards, across LG comparisons were done with a Kruskal−Wallis test and a *post hoc* Wilcoxon test as described above.

## 3. Results

### (a) Elevated levels of male-biased gene expression

**Figure 2.**
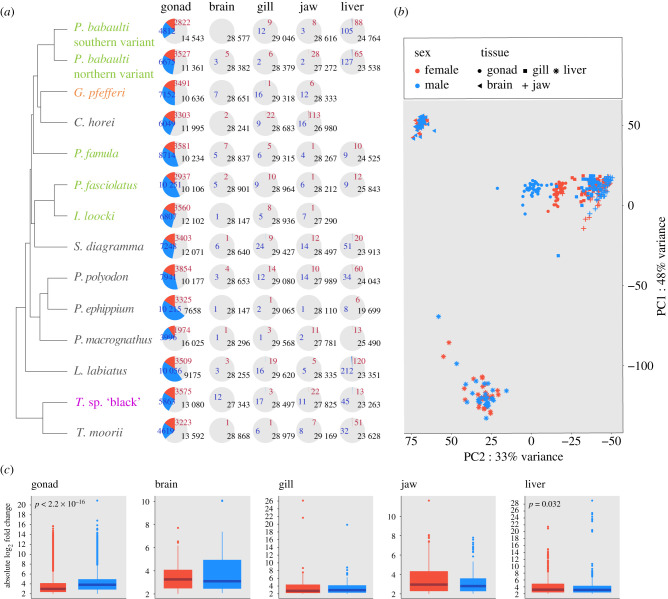
(*a*) Proportions of sex-biased genes within each organ and species are indicated with pie charts along the species tree (blue: MBGs; red: FBGs; grey: unbiased genes). Species names are coloured with respect to sex chromosomal systems as depicted in figure 1. For three species (*G. pfefferi, C. horei* and *I. loocki*) not enough replicates per sex were available to investigate sex-biased gene expression in the liver. These species were hence excluded. (*b*) Principal component analysis of gene expression across organs and samples. Samples are coloured according to sex (blue: males; red: females), symbol shapes refer to organ type (see figure inlet). Proportion of variance explained by the first two principal components (PC1 and PC2) is indicated at the plot axes. (*c*) The plots summarize the absolute change in gene expression for female (red) and male (blue) biased genes across species within each organ. Boxplot centre lines represent the median, box limits the upper and lower quartiles and whiskers the 1.5× interquartile range. Outliers are not shown.

Our analysis of SBG expression within the different organs revealed—as expected—a consistently large number of SBGs in the gonads (27.2–63.9% of expressed genes) and a much lower (if any) number (0.1–1.4%) in the somatic organs (figure 2; electronic supplementary material, figures S2–S5).

Within species, the fractions of MBGs and FBGs in the gonads were relatively constant across reference LGs with more MBGs than FBGs throughout (electronic supplementary material, figure S3; note that LG3a and LG3b showed consistently lower levels of SBGs; however, these chromosomes are enriched in repetitive sequence content in the reference genome and have overall lower mapping rates). Analysed across all chromosomes, we noted a depletion of MBGs compared to FBGs on LG19, which was significantly strongest for this LG in particular in the LG19 XY species *T.* sp. ‘black’ (*p* = 3.22 × 10^−12^) and also pronounced in several other species (*I. loocki*, *P. macrognathus*, *P. babaulti* southern variant, *P. babaulti* northern variant, *P. polyodon*, *P. fasciolatus*, *P. ephippium*, *L. labiatus, S. diagramma* and *G. pfefferi*).

Although the somatic organs showed few SBGs, here in contrast to the gonad, in the XY-19 species *T.* sp. ‘black’, the majority of MBGs in the brain, gill and jaw were indeed located on LG19 and on LG15 in the LG11/LG15 XY species *G. pfefferi* (electronic supplementary material, figure S4). Of those MBGs, two genes (*adgdr2, adhesion G-protein-coupled receptor D2* and LOC109194780, an ncRNA) were present in three organs of *G. pfefferi* and one (*impg1*, *interphotoreceptor matrix proteoglycan 1*) of *T.* sp. ‘black’ (table 1).

**Table 1.** Somatic MBGs located on sex chromosomes of *G. pfefferi* (LG15) and *T*. sp. ‘black’ (LG19).

species	linkage group	tissue	gene ID	gene name
*G. pfefferi*	LG15	brain	LOC102082324	*adhesion G-protein-coupled receptor D2*
			LOC109194780	uncharacterized ncRNA
			LOC102080291	*survival motor neuron protein*
			LOC100704264	*tyrosine-protein kinase JAK2*
		gill	LOC100708389	*protein cornichon homolog 3*
			LOC102082324	*adhesion G-protein-coupled receptor D2*
			LOC109194780	uncharacterized ncRNA
			LOC100700205	*intestinal-type alkaline phosphatase*
			LOC100699936	*alkaline phosphatase*
			LOC102080084	*transcription initiation factor TFIID subunit 4*
			LOC100708082	*collagen alpha-1(XII) chain*
		jaw	LOC100708389	*protein cornichon homolog 3*
			LOC109194780	uncharacterized ncRNA
			LOC102082324	*adhesion G-protein-coupled receptor D2*
			LOC100700205	*intestinal-type alkaline phosphatase*
			LOC102080291	*survival motor neuron protein*
*T*. sp. *‘*black’	LG19	brain	LOC102078936	*interphotoreceptor matrix proteoglycan 1*
			LOC100695983	uncharacterized mRNA
			*kif15*	*kinesin family member 15*
			*ddo*	*D-aspartate oxidase*
			*foxg1*	*forkhead box G1*
		gill	LOC100711378	*coiled-coil domain-containing protein 177*
			LOC102078223	uncharacterized ncRNA
			LOC102078936	*interphotoreceptor matrix proteoglycan 1*
			LOC102082307	*tudor domain-containing protein 6*
			LOC100708336	*unconventional myosin-X*
			LOC100695242	*dehydrogenase/reductase SDR family member 7*
			LOC100708898	*neuroglobin*
			LOC100695769	*leucine-rich repeat-containing protein 9*
		jaw	LOC102082307	*tudor domain-containing protein 6*
			LOC100695242	*dehydrogenase/reductase SDR family member 7*
			LOC100708336	*unconventional myosin-X*
			LOC102078936	*interphotoreceptor matrix proteoglycan 1*
			LOC100708898	*neuroglobin*
			*tpo*	*thyroid peroxidase*

Across all species, in addition to having consistently more FBGs than MBGs (figure 2*a*), the gonads also had significantly higher differences in gene expression levels for MBGs than FBGs, whereas most other organs showed the opposite trend (figure 2*c*).

On the species level, there was little variation among LGs in the degree of sex bias in the gonads (electronic supplementary material, figures S5 and S6). We noted a significant decrease of male bias on LG19 solely in one of the LG5/LG19 XY species (*P. babaulti* northern variant with a similar trend also on LG5) and a significant increase of male bias on LG15 in the LG11/LG15 species *G. pfefferi*.

### (b) High degree of sex-biased gene expression turnover

**Figure 3.**
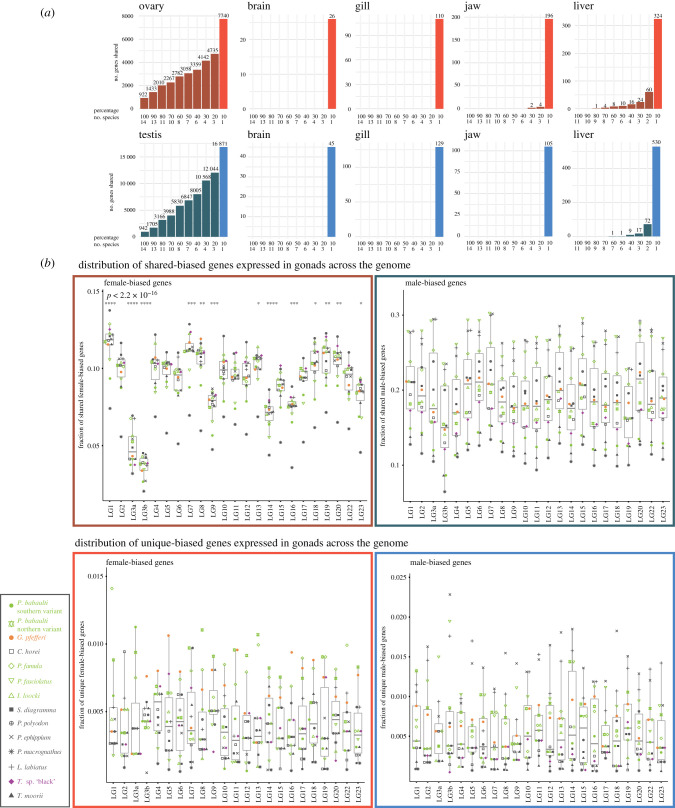
(*a*) Barplots indicate the number of unique and shared sex-biased genes across species and organs (red: female-biased; blue: male-biased). Number on bars indicates the number of genes in each category. (*b*) Upper panel: chromosomal distribution of genes with sex-biased expression in the gonad in at least two species; lower panel: chromosomal distribution of genes with species-specific sex-biased expression in the gonad (red: ovary; blue: testis). Boxplot centre lines represent the median, box limits the upper and lower quartiles and whiskers the 1.5× interquartile range. Per species values are indicated with symbols depicted in the inlet. Symbols indicating statistical significance are *p ≤ 0.05, **p ≤ 0.01, ***p ≤ 0.001, ****p ≤ 0.0001.

Within organs across species, the level of FBGs and MBGs that we could detect as shared across species was relatively low (FBGs: gonad 42%, brain 0%, gill 1%, jaw 11%, liver 16%; MBGs: gonad 45%, brain 4%, gill 6%, jaw 0%, liver 13%; figure 3*a*; electronic supplementary material, figure S7), suggestive of high levels of SBG turnover.

This analysis further revealed that the depletion of MBGs on LG19 is a shared feature across species since there was no decrease but rather an increase in species-specific MBGs on LG19 (figure 3*b*), while MBGs shared across species showed the decrease of MBGs that we noted already on the species level (figure 3*b*; electronic supplementary material, figures S3 and S4). LG19 is further enriched in shared FBGs compared to other LGs but not in species-specific FBGs.

For gonads, overall the distribution on chromosomes appeared more variable in shared FBGs than MBGs, which were rather evenly distributed along the genome (figure 3). Yet, the levels of expression were more variable for MBGs than FBGs (electronic supplementary material, figure S3).

Both the shared gonad FBGs and MBGs were enriched for a functional annotation in ‘reproductive process' supporting their predominant role in reproduction. Shared FBGs were further significantly associated with ‘cell division’ and ‘(lipid) biosynthetic processes' and shared MBGs with several hormonal pathways (electronic supplementary material, figure S8).

Also, within a species, there was little overlap of SBGs across organs (electronic supplementary material, figures S9 and S10). Within a species, only *P. famula* and *T.* sp. ‘black’ had each a single gene that was female-biased across all organs (LOC100702510, *interferon-induced very large GTPase 1-like* and LOC100699357 *major histocompatibility complex class I-related gene protein-like*, respectively), and *P. fasciolatus* and *G. pfefferi* had each two MBGs that were male-biased across all organs (uncharacterized protein LOC109194565 and LOC102076543 *polymeric immunoglobulin receptor* for *P. fasciolatus* and uncharacterized protein LOC109194780, LOC102082324 *adhesion G-protein-coupled receptor D2* for *G. pfefferi,* see above).

In gonads, gene expression levels in females were more conserved than in males, whereas the opposite pattern was observed in the liver (electronic supplementary material, figure S2). Overall gene expression similarity was dominated by phylogenetic relationships also in SBGs (electronic supplementary material, figure S2).

**Figure 4.**
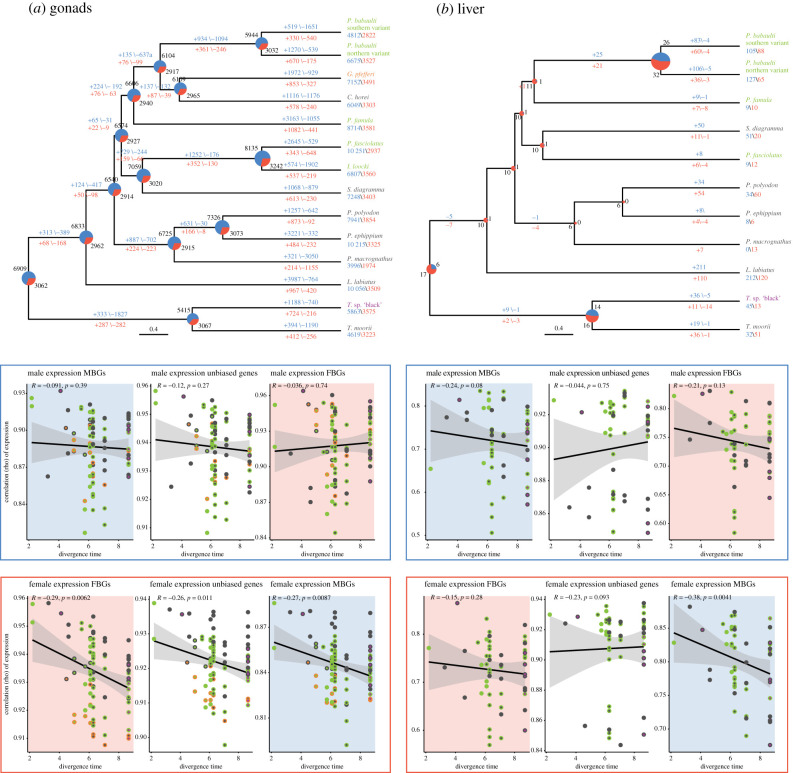
Evolution of sex-biased gene expression in gonads (*a*) and liver (*b*). Ancestral state reconstructions for sex-biased gene expression are shown along the species tree in gonads and liver. Pie charts indicate the number of MBGs (blue) and FBGs (red) reconstructed at nodes, numbers in blue above edges indicate gain and loss of MBGs and numbers in red below edges indicate gain and loss of FBGs. Pairwise Spearman's rank correlation coefficients for male gene expression (blue box) and female gene expression (red box) as a function of their divergence times [11] is depicted in the categories MBG (blue background), FBG (red background) and unbiased (white background) for gonads and liver. Pairwise species comparisons are plotted with two coloured symbols which refer to each species sex chromosome as depicted in figure 1 together with the regression line (black) and 95% confidence interval (grey).

To further investigate the dynamics of SBG turnover along the species' history, we reconstructed ancestral sex bias for the organs with the highest amount of shared SBGs, gonads and liver (figure 4). In agreement with the present-day data, this supported higher levels of MBGs as FBGs throughout evolutionary time in gonads.

The reconstructed amount of SBGs in the liver was low with consistently more FBGs than MBGs and higher levels of gains of MBGs than FBGs in the terminal branches. Likewise, in the gonads, the terminal branches were dominated by gains of MBGs, however not exclusively. In concordance with the absolute numbers, gains of FBGs in the terminal branches were lower than of MBGs. Although the absolute proportion of FBGs and MBGs in gonads remained relatively constant over time, there were consistently high levels of SBG turnovers; in contrast with the terminal branches, however, with a similar amount of losses and gains within FBGs and MBGs, respectively.

In concordance with the PCA analysis (figure 2; electronic supplementary material, figure S2), there was no significant phylogenetic signal in male gene expression (figure 4) in the gonad with overall lower correlations of male gene expression than female gene expression across species while female expression showed clear phylogenetic correlation. In the liver, we observed a similar pattern in male expression (no significant correlation with divergence), however, here also FBGs as well as unbiased genes in females did not show a strong phylogenetic signal in female expression of FBGs. As in the gonads, MBGs in females showed a significant correlation with phylogenetic distance with overall lower levels of correlation. Interestingly, unbiased genes showed overall very little phylogenetic signal (only significant in females in the gonad). Across all tissues, female expression showed stronger phylogenetic signal than male expression (electronic supplementary material, figure S11).

The number of SBG turnovers in the gonad and liver did not show a significant association with levels of sexual selection (electronic supplementary material, figure S12) nor with the degree of sex chromosome differentiation (electronic supplementary material, figure S12).

### (c) Rates of gene expression evolution

**Figure 5.**
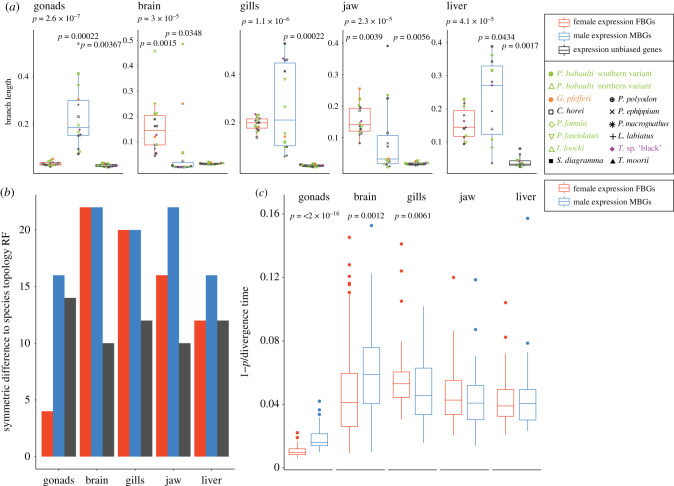
(*a*) Boxplots show cumulative branch length from root to tips of expression phylogenies of FBGs in females (red) and MBGs in males (blue) and unbiased genes for each organ. Boxplot centre lines represent the median, box limits the upper and lower quartiles and whiskers the 1.5× interquartile range. Per species values are indicated with symbols depicted in the inlet. (*b*) Robinson–Foulds (RF) distance of expression phylogenies of each organ to topology of the species tree [11]. (*c*) Rate of expression changes assessed as [1 – *ρ*]/divergence time [11] for FBGs and MBGs in each organ.

The investigation of turnovers of SBGs indicated fast evolution of sex-biased expression in Tropheini species. This pattern is supported by gene expression phylogenies (figure 5*a*), which showed that across all organs, SBGs have longer and more variable branch lengths, i.e. faster and divergent accumulation of expression divergence than unbiased genes. FBGs showed a trend for higher levels than MBGs in the brain and jaw while MBGs evolved faster than FBGs to some extent in the liver but most drastically so in the gonads. Concordantly, the expression phylogeny of FBGs in the gonad was also most similar to the species tree topology (figure 5*b*) consistent with the strong phylogenetic signal in FBG expression correlations (figure 4). In the other organs, unbiased phylogenies were consistently closest to the species topology and again MBGs showed less phylogenetic signal than FBGs in the gonad and also in the liver and jaw.

In expression phylogenies of gonad SBGs of each LG separately, MBGs in the gonad consistently had significantly higher levels of expression divergence than unbiased genes and mostly also as FBGs, including the sex-linked LGs 5, 22, 15 and 19 (electronic supplementary material, figure S13). This difference was less pronounced for FBGs compared to unbiased genes. In the liver, in which the genome-wide trend showed somewhat higher divergence for MBGs than FBGs in some but not all species and longer branch length in both FBGs and MBGs than unbiased genes, we detected on the chromosome level much less pronounced divergence in MBGs than FBGs than in the gonads. Yet here again, the sex chromosomes LG15 and LG19 showed higher divergence in MBGs than FBGs.

Concordantly, in gonads and also in the brain, MBGs showed a significantly higher rate of expression changes than FBGs (figure 5*c*), while the opposite was true in the gill and no significant difference was found for the liver and the jaw.

We next tested if correlations of male and female expression levels differed by chromosome. None of the sex chromosomes showed a decrease of pairwise gene expression correlations across all species pairs (electronic supplementary material, figure S14).

To identify driving forces of gene expression divergence among recently diverged species, we first calculated Δ*x*, which assesses gene expression divergence among species relative to expression variance within a species. We identified a very small fraction of SBGs (between 0 and 5%) with signatures of directional selection according to Moghadam *et al.* [42], i.e. Δ*x* > 1 or Δ*x* < 1 (electronic supplementary material, figure S15). Overall, we identified more genes under putative positive selection to be over- than under-expressed.

### (d) Evolutionary rates of sex-biased genes

The evolutionary rate of expression of SBGs in Tropheini cichlids was elevated throughout the genome and the oldest sex chromosome, LG19, showed signs of depletion of MBGs. Expression levels seemed to largely be evolving under drift, however, somewhat rapidly. We next aimed to investigate if SBGs also showed increased evolutionary rates on the sequence level and if those also differed between MBGs and FBGs.

**Figure 6.**
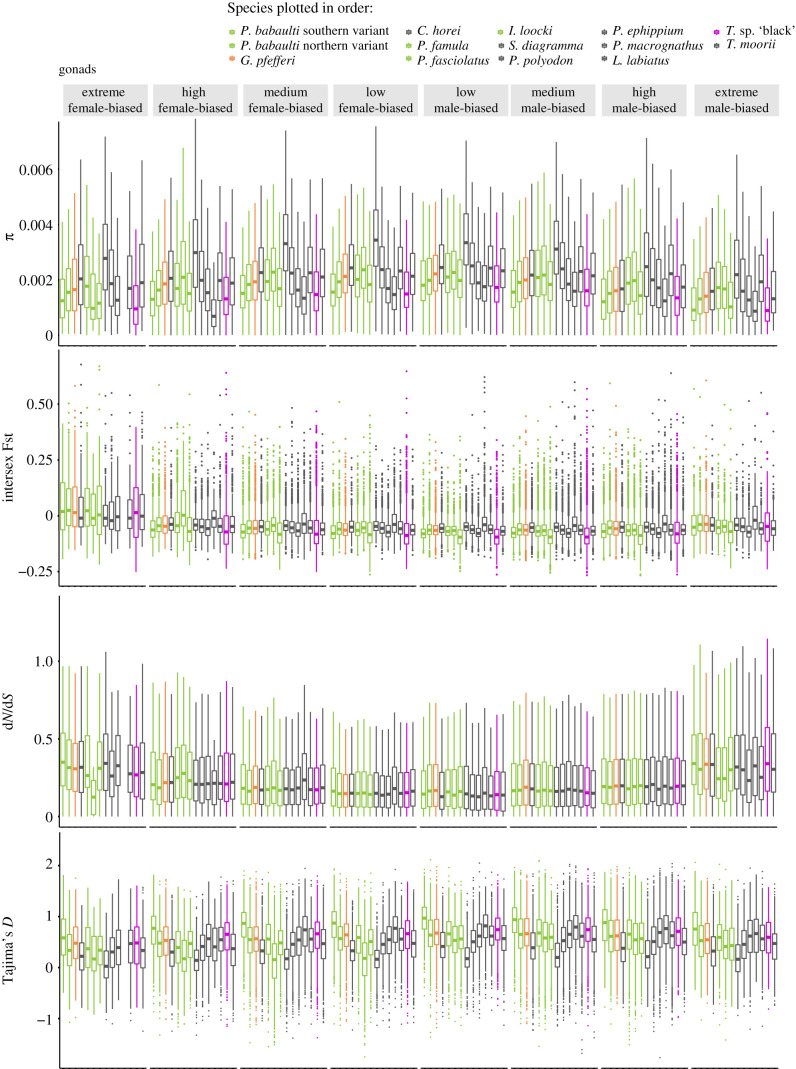
Boxplots depict population statistics per species in the gene expression categories extreme, high, medium and low female- and male-biased for genes expressed in gonads. Boxplot centre lines represent the median, box limits the upper and lower quartiles and whiskers the 1.5× interquartile range. Outliers are not shown for d*N*/d*S* and *π*.

Focusing on the organs with the highest amount of sex bias allowing for comparisons across LGs, we analysed SBGs in the gonad and liver. Overall, levels of nucleotide diversity (*π*) within species did not depend on levels of sex bias nor sex (figure 6; electronic supplementary material, figure S16)

In contrast with *π*, we detected an overall increase of between-sex sequence differentiation with increasing sex bias of expression. This pattern was more pronounced in FBGs, which showed overall higher intersex Fst levels than MBGs. We observed this increase in male–female differentiation with increase in sex bias in both organs (figure 6; electronic supplementary material, figure S16).

Likewise, we noted increasing levels of d*N*/d*S* with increasing sex bias; however, here in contrast with intersex Fst, MBGs overall showed higher levels than FBGs in the gonad and the opposite pattern was detected in the liver (figure 6; electronic supplementary material, figure S16).

Comparing the SBGs with an expression pattern evolving under putative positive selection to those that on the sequence level also showed increased levels of d*N*/d*S* returned 10 genes in gonads (two of them shared in two species) and one in the liver. These genes had functions in immune system response, steroid synthesis and cell metabolism (table 2).

**Table 2.** Genes under putative positive selection identified by Δ*x* and d*N*/d*S*.

tissue	species ID	location	gene	gene name
gonads	*P. babaulti* (southern variant)	LG10	*grxcr2*	*glutaredoxin and cysteine-rich domain-containing 2*
	*G. pfefferi*	LG23	LOC100706322	*potassium voltage-gated channel subfamily H member 8*
	*G. pfefferi*	LG9	*lrrc19*	*leucine-rich repeat-containing 19*
	*G. pfefferi*	LG19	LOC100700106	*rho-related GTP-binding protein RhoV*
	*G. pfefferi*	LG13	LOC102078314	uncharacterized
	*C. horei*	LG22	LOC100711324	*interleukin-11*
	*C. horei*	LG7	LOC109202857	*plancitoxin-1-like*
	*P. fasciolatus*	LG4	LOC100701173	*cytochrome c oxidase subunit 6A, mitochondrial*
	*C. horei*	LG6	*qprt*	*quinolinate phosphoribosyltransferase*
	*S. diagramma*	LG7	LOC109202857	*plancitoxin-1-like*
	*C. horei*	LG16	*hsd3b1*	*hydroxy-delta-5-steroid dehydrogenase, 3 beta- and steroid delta-isomerase 1*
	*P. macrognathus*	LG7	LOC109202857	*plancitoxin-1-like*
	*L. labiatus*	LG7	LOC109202857	*plancitoxin-1-like*
	*L. labiatus*	LG9	*lrrc19*	*leucine-rich repeat-containing 19*
liver	*L. labiatus*	LG14	LOC100694080	*transmembrane protease serine 4*

Overall levels of Tajima's *D* were supportive of balancing selection in FBGs and MBGs. MBGs had in general higher values than FBGs with a drop in both categories in the most extremely biased genes, agreeing with the increase of d*N*/d*S* in this category of genes (figure 6).

### (e) Sequence evolution of sex chromosomes

**Figure 7.**
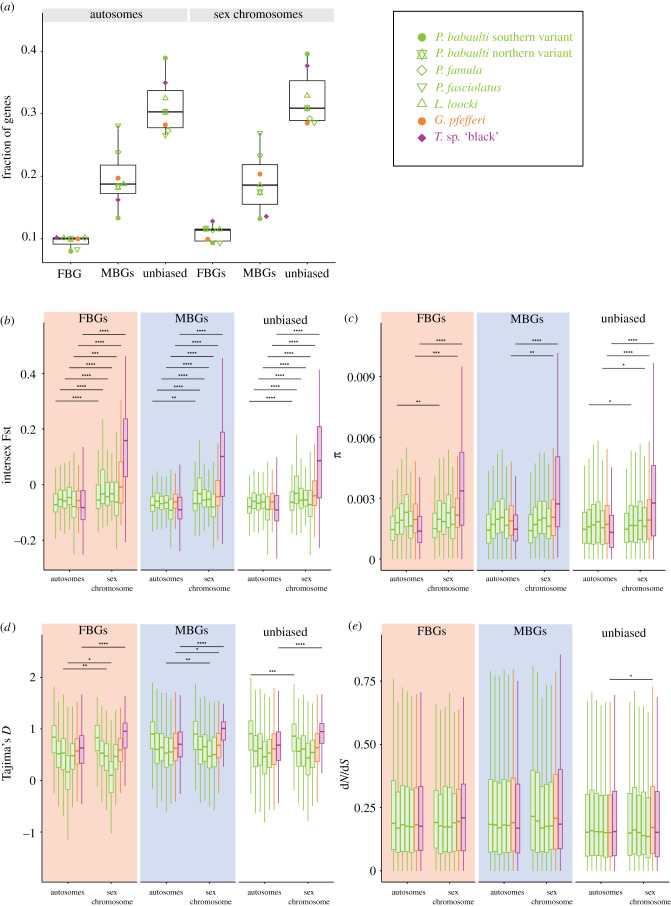
Comparisons of gonad sex-biased and unbiased genes on autosomes and the respective sex chromosomes of the seven taxa with previously described sex chromosomes. Colours refer to sex chromosomes as shown in figure 1: green, LG5/LG19; orange, LG11/LG15; and purple, LG19. (*a*) Fractions of FBGs, MBGs and unbiased genes. Symbols of individual datapoints refer to species as depicted in the inlet. (*b*) Intersex Fst of FBGs, MBGs and unbiased genes. (*c*) *π* of FBGs, MBGs and unbiased genes. (*d*) Tajima's *D* of FBGS, MBGs and unbiased genes. (*e*) d*N*/d*S* of FBGs, MBGs and unbiased genes. Species values in (*b*)–(*e*) are plotted in the order shown in the inlet. Boxplot centre lines represent the median, box limits the upper and lower quartiles and whiskers the 1.5× interquartile range. Significance levels of a Wilcoxon test comparing for each expression category autosomes and sex chromosomes are indicated with *p ≤ 0.05, **p ≤ 0.01, ***p ≤ 0.001, ****p ≤ 0.0001. Outliers are not shown for (*b*–*e*); not significant *p*-values are not shown.

While there was no over-representation of gonad SBGs *per se* on the sex chromosomes (see above and figure 7*a*, decrease of MBGs on the sex chromosome of some species, increase in others), the sex chromosomes showed on the sequence level typical signs of sex chromosome evolution regardless of sex bias reflected by increased levels of intersex Fst (figure 7*b*).

When analysed within species across individual chromosomes, in particular LG19 in *T*. sp. ‘black’ showed increased nucleotide diversity and intersex Fst for both FBGs and MBGs and unbiased genes in gonads and some extent liver (figure 7 comparing the sex chromosome to all autosomes combined; electronic supplementary material, figures S17–S20 for single chromosome analysis). Intersex Fst as well as π on *T.* sp. ‘black’ LG19 were higher for FBGs than MBGs in gonads (*p* = 0.0014 for Fst, not significant for *π*). The much younger sex chromosome of *G. pfefferi* (LG11/LG15) also showed elevated levels of intersex Fst and *π*, again in particular in FBGs but also in MBGs in the gonad (*p* = 5.2 × 10^−5^ for Fst when compared across all LGs, not significant for *π*; see also figure 7).

In the LG5/LG19 XY species, the sex chromosomes did not significantly differ in nucleotide diversity with the only exception of increased *π* in FBGs on LG19 of *P. babaulti* northern variant (electronic supplementary material, figures S17 and S18, figure 7, when all autosomes are combined *π* was also significant higher in unbiased genes on the sex chromosome).

In all but one species that have lost the LG19 XY system, namely in MBGs in *P. ephippium*, intersex Fst values on LG19 were not elevated anymore. However, in the species that transitioned to the LG5/LG19 system which does not show sex linkage over the full length of LG19 but only in the last approximately 7 Mb [13], LG19 and LG5 showed elevated intersex Fst in MBGs in *P. famula*, *P. fasciolatus*, *I. loocki* and *P. babaulti* northern variant (*P. babaulti* southern variant showed elevated intersex Fst only on LG19 in MBGs; all species showed significantly increased Fst when the sex chromosome was compared to all autosomes combined for Fst in FBGs, MBGs and unbiased genes; figure 7*b*). FBGs also showed elevated intersex Fst in all LG5/LG19 XY species but *I. loocki* (similar trend, however not significant). Overall, Fst values in gonads and liver were significantly higher for FBGs (*p* < 2 × 10^−16^ and *p* = 5.3 × 10^−5^, respectively), but not in gills or jaw. In the brain, MBGs had higher intersex Fst values (*p* = 0.02).

The degree of increased intersex Fst and somewhat of *π* when compared between all autosomes combined and the sex chromosome reflects the degree of sex chromosome differentiation, with more pronounced sex-chromosome–autosome differences in the species with more strongly differentiated sex chromosomes (figure 7*b,c*).

When analysed on a per chromosome basis, Tajima's *D* was significantly decreased in gonad FBGs on LG19 in two of the LG5/LG19 species (*P. fasciolatus* (*p* = 0.023), *P. famula* (*p* = 0.03040)) and drastically increased in FBGs in the LG19 species *T.* sp. ‘black’ (*p* = 1.4 × 10^−15^). Note that it was also significantly decreased in the sister species *T. moorii* (*p* = 0.00781) as well as in *P. polyodon* (*p* = 0.010) and *C. horei* (*p* = 0.0138), all species for which no sex chromosome was identified (electronic supplementary material, figure S21, no significant difference for liver SBGs electronic supplementary material, figure S22).

In gonad MBGs, Tajima's *D* was again drastically increased on LG19 in the LG19 species *T.* sp. ‘black’ and otherwise decreased (significantly in three of the XY LG5/LG19 taxa *P. babaulti* northern variant, *I. loocki*, *P. fasciolatus* and also in the species without sex chromosomes*, C. horei*, *S. diagramma*, *P. polyodon*, *P. ephippium*, *L. labiatus*, *T. moorii*). When focusing only on the species for which sex chromosomes were identified comparing the sequence evolution of the sex chromosome to all autosomes combined, the same signals were visible with a notable increase of Tajima's *D* on LG19 in *T.* sp. ‘black’ regardless of sex bias (figure 7*d*).

Within species, d*N*/d*S* did not significantly differ among chromosomes in FBGs nor MBGs, when chromosomes were compared separately (electronic supplementary material, figure S23). Likewise, there was solely a significant increase in d*N*/d*S* in unbiased genes of *G. pfefferi* when comparing the sex chromosomes with all autosomes combined (figure 7*e*).

## 4. Discussion

Sex chromosomes as well as differential gene expression define and maintain the sexes of a species. This has mainly been studied in species with well-differentiated sex chromosomes that have been subject to sex-specific selection forces for longer periods of time. We here set out to study the interplay of sex differences in gene and sequence evolution in an array of closely related species that diverged recently and have different sex chromosomes.

Across the 14 taxa investigated, we detected a large amount of SBGs in gonads, as expected given the structural and functional differences between the testis and ovaries. Agreeing with previous observations in cichlids [53], we detected much less if any SBGs in the soma. Similar patterns have been described from other species (e.g. [44,54–56]), still, the amount of SBGs in the somatic organs of the here studied cichlid species seems particularly low. We suspect that sex bias in gene expression might be higher during development or in other organs not included here, accounting for the extensive sexual dimorphism observed in many cichlids. Alternatively, phenotypic differences could rely on less pronounced gene expression differences that we might be unable to detect with the sequencing data at hand or that more generally fail the commonly used thresholds defining differential gene expression.

In the gonads, we detected more MBGs than FBGs and the data at hand suggest the opposite trend in the somatic organs. The opposing pattern was reported for flycatchers [54]. Yet, more FBGs than MBGs in somatic organs with higher levels of sex bias have previously also been described in frogs [57] and butterflies [44]. Our data could further support that sex-specific selection differs among organs within a species but also across species. A possible explanation is that, in cichlids, selection on males could be more pronounced in gonads while being higher in females in the other organs. We assume that sex-specific selection in cichlids affects the number of genes that show sex bias as well as the degree of sex bias, similar to observations in frogs [57]. This pattern will require further in-depth study of traits under sex-specific selection and their underlying gene expression network.

Sex bias in gene expression is often interpreted as a sign of ongoing sexual conflict. A resolution of conflict can be achieved by the location of sexually antagonistic genes on sex chromosomes, also a supposed major driving force in the emergence of new sex chromosomes [58,59]. Such a resolution of sexual conflict seems evident in species with differentiated sex chromosomes and is reflected by an accumulation of FBGs (feminization) on X chromosomes and MBGs (masculinization) on Z chromosomes (reviewed in [60]). In agreement with recent data from species with young/little-differentiated sex chromosomes [57], we did not observe an accumulation of SBGs on the sex chromosomes of cichlids, at least not SBGs of gonads, the organs with the highest degree of sex bias. In contrast with other XY species, we rather noted a demasculinization (or equal amount of feminization and masculinization) than feminization on the oldest here investigated sex chromosome (LG19), which somewhat persisted even after the return of this sex chromosome to an autosome.

Owing to our species set-up, we could further disentangle this pattern, which suggests a depletion of generally conserved ‘testis–genes’ on LG19 with a simultaneous increase of genes with a conserved ovary-overexpression, which persists independent of sex chromosomal status and thus implies a selective constraint or could reflect the past history of this LG as a sex chromosome. When focusing on species-specific, i.e. recently acquired gonad SBGs, rather a masculinization of LG19 was detectable supportive of a predominant role in species-specific resolutions of sexual conflict.

The somatic SBGs, however, point towards a masculinization of cichlid sex chromosomes, for LG19 in the XY species *T.* sp. ‘black’ and notably also for LG15 in the much younger XY system on LG11/LG15 in *G. pfefferi*, again supporting differing selection pressures across organs.

Across the Tropheini cichlids studied, the age of the sex chromosome does not seem to be important for this process of masculinization but rather the size of the sex-linked region. *Gnathochromis pfefferi* and *T.* sp. ‘black’ show signs of sex chromosome differentiation across the entire LG lengths suggestive of large regions with suppressed recombination. The sex-linked region is much smaller in the XY LG5/LG19 taxa [13] without a notable masculinization in SBG distribution.

Across species, we noted that although there was not a general accumulation on sex chromosomes, the distributions of FBGs and MBGs differed along the genome. The distribution of FBGs varied more across the genome, while MBGs were rather evenly spread across chromosomes but showed more variation in their expression levels than FBGs. The amount of shared MBGs and FBGs across species was, however, very similar.

We did not find evidence that LGs, which became sex-linked throughout the evolution of Tropheini, were enriched in SBGs prior to their recruitment as a sex chromosome. This suggests that changes on the sequence level of Tropheini sex chromosomes predate evolutionary changes in sex-specific gene expression levels.

Cichlids of Lake Tanganyika show impressive levels of phenotypic and ecologic diversity that evolved within less than 10 Myr [11,61]. The turnover rate of sex chromosomes holds pace with this rapidity of cichlid evolution [13]. Likewise, our ancestral state reconstructions for sex bias in gonads and liver support high levels of SBG turnovers with rather consistent levels of SBG gains in gonads within the last 5 Myr of evolution of the here studied Tropheini lineage and in particular in the terminal branches. Turnover in the liver happens almost exclusively on the terminal branches and is dominated by changes in MBGs.

A very similar evolutionary scenario with high gains of SBGs throughout and in particular on the terminal branches has previously been described in an avian clade (Galloanserae) [62], which however with 90 Myr is much older than the here studied Tropheini (approx. 5 Myr).

Because SBG expression is supposed to be the major source of sex differences on the phenotypic level, it is broadly assumed that sexual selection shapes sex bias in gene expression. In agreement with this, Galloanserae indeed show increased rates of MBG turnovers with increasing levels of phenotypic proxies for sexual selection [62]. Sexual selection is certainly a prominent driver in cichlid evolution (e.g. [18,63]). However, in contrast with Galloanserae, we did not detect a correlation between the levels of sexual selection and the number of SBG turnovers. In agreement with this, gene expression of SBGs in Tropheini largely evolves under drift, similar to SBGs in butterflies [44]. Furthermore, the degree of sex chromosome differentiation had also no impact on SBG turnovers.

Overall, gene expression evolution shows a strong phylogenetic signal in Lake Tanganyika cichlids resembling patterns in mammals albeit much shorter evolutionary distances among species [17]. We detected the same signal in female expression with a strong negative correlation between expression similarity and divergence time, in particular in the ovary. Male expression in testis on the other hand did not show this connection and evolved more rapidly than female gonad expression, which showed a particularly strong phylogenetic signal compared to other organs. FBGs compared to MBGs outside the gonads, however, showed higher or similar levels of expression divergence and not much higher if any levels of phylogenetic signals as MBGs. This suggests that different organs evolve with different speeds and agreeing with observations from other animal groups, testis is evolving fastest (e.g. [22]). Our multi-species multi-organ analysis shows that this is not driven by sex *per se* but that the organ type can have a large impact as well.

Studies in XY species with differentiated sex chromosomes such as *Drosophila* and mammals have shown higher expression divergence on the X chromosome, a ‘fast-X-effect’ not only in sequence evolution but also in gene expression (reviewed in [64]). In contrast with this, we did not detect signs of faster expression divergence on any of the sex chromosomes, not across species nor within species pairs that share the same sex chromosome.

Likewise, when analysing sequence evolution of the sex chromosomes and similar to the young sex chromosomes in frogs [57], we found no strong indications for a ‘fast-X effect’ with levels of d*N*/d*S* not differing between-sex chromosomes and autosomes.

Yet, sequence evolution on the sex-linked LGs clearly differed from the autosomes. We detected some of the typical signs of sex chromosome differentiation with elevated intersex Fst on all sex chromosomes (more pronounced in the species with stronger differentiated sex chromosomes) and increased nucleotide diversity on the most strongly differentiated and oldest sex chromosome, LG19 in *T.* sp. ‘black’ and to some extent also on LG11/LG15 in *G. pfefferi*. While this suggests accumulating allelic difference between X and Y resulting from suppressed recombination, a lack of dosage compensation and/or the depletion of MBGs and the still high amount of genes shared between X and Y could account for the seeming absence of a fast-X effect [65].

We also noted a significant increase of Tajima's *D* on LG19 in *T.* sp. ‘black’ whereas LG5 and LG19 showed decreased Tajima's *D* in several of the XY LG5/LG19 species. Because LG19 in *T.* sp. ‘black’ also showed increased nucleotide diversity and intersex Fst reflecting the sequence divergence between sex-linked (X and Y) alleles, this might also account for increased Tajima's *D*. The XY LG5/LG19 species also showed sequence differentiation on the sex chromosomes (higher intersex Fst) but to a lower extent and not captured by *π*. The decrease in Tajima's *D* in these younger sex chromosomes could indicate different selective forces acting at different stages of sex chromosome evolution. In *G. pfefferi*, which has sex chromosomes that are even younger but intermediate in their degree of sex chromosome differentiation between *T*. sp. ‘black’ and the XY LG5/19 species, Tajima's *D* on the sex chromosomes did not much differ compared to autosomes (elevated in MBGs only). We noted the decrease of Tajima's *D* on LG19 also in species that lost the XY LG19 system but do also not have the XY LG5/LG19 system, which suggests that after returning to an autosome (supposedly by losing the Y chromosome), the previously X-linked sequences on LG19 (still) show(ed) reduced levels of nucleotide diversity.

The here studied sex chromosomes all showed signs of sex chromosome sequence differentiation and the two more differentiated sex chromosomes contain on the one hand conserved MBGs in the somatic organs—probably reflecting resolved sexual conflict—and, on the other hand, are feminized/de-masculinized by a depletion of testis–genes in the gonadal tissue.

Overall, however, the here studied sex chromosomes do neither disproportionally contribute to the establishment of sexual dimorphism nor does SBG turnover seem to be driven by sexual selection. It rather seems that sequence divergence between X and Y predates expression divergence of the sex chromosomes among the sexes. We did not find any support for an accumulation of SBGs on chromosomes prior to their recruitment as sex chromosome nor after. The rather even distribution of SBGs across chromosomes certainly facilitates the high turnover rate of sex chromosomes generally observed in Lake Tanganyika cichlids [13].

Our genome-wide sequence analysis of SBGs revealed an association of sex bias in expression with patterns of sequence diversity across recently diversified species. Theory predicts that sexual antagonism impacts sequence evolution differentially in the two sexes [1,66] and SBG expression is often interpreted as an indicator of (hotly debated if ongoing or resolved) sexual antagonism. In contrast with other recent studies [54,67], we did not see increased sequence diversity *per se* with increasing sex bias in gene expression.

Yet, we found increasing levels of signatures of positive selection (d*N*/d*S*) with increasing sex bias in both FBGs and MBGs, with higher levels in MBGs. Increased positive selection coupled with fast rates of evolution has been shown before for testis (e.g. [68,69]) and MBGs [54]. A strikingly similar pattern with evidence for relaxed purifying selection in MBGs and FBGS has recently been described in seed beetles [67] and a similar tendency of increasing d*N*/d*S*, particularly with increasing male bias, was also detected in birds [62]. However, here supposedly not driven by the often assumed underlying sexual selection as source for positive selection but rather resulting from non-adaptive genetic drift.

Similar to seed beetles, levels of balancing selections in the strongest SBGs were reduced in Tropheini, further corroborating that strong sex bias towards sex-limited expression resolves sexual conflict over gene expression [70]. We also observed the same trend for increased balancing and hence potentially sexually antagonistic selection with decreasing sex bias as described in seed beetles, albeit in our case similarly in MBGs and FBGs and with higher values in MBGs than FBGs. In seed beetles, by contrast, intermediate MBGs rather showed signs of purifying selection.

Closely resembling the pattern of positive selection, intersex Fst also increased with increasing sex bias, although here with overall higher values in FBGs than MBGs. Increased intersex Fst is often interpreted as a signature of sexual conflict in particular over survival, whereas an increase solely in Tajima's *D* rather reflects conflict over reproduction (reviewed in [39]).

In our case, when comparing across all species, Tajima's *D* was lowest when intersex Fst was highest, i.e. in extreme FBGs and MBGs, albeit with higher Fst levels in FBGs. This particular pattern has been interpreted to rather reflect difference in sex-specific viability than sexual conflict [36,39]. Why in cichlids this would be more pronounced in FBGs remains to be investigated. Taken together, the observed patterns of sex bias could support resolved conflict in strongly biased genes in particular in FBGs, which has previously rather been demonstrated for MBGs ([39] and references therein).

The here studied Tropheini cichlids consistently showed the same patterns of sequence evolution of SBGs, although they had constantly high levels of SBG turnovers. This suggests that on the sequence level, similar evolutionary forces impact SBGs within species, whereas the actual conflict about them varies across species that diverged very recently.

## Data accessibility

All primary data used in this paper have been published elsewhere and reference is given in the main text.
